# Supplementary material for: Oxidative Stress and Antioxidant Therapies in Friedreich’s Ataxia
Source: Cells. 2025 Sep 9;14(18):1406. doi: 10.3390/cells14181406 (PMC12469045; doi:10.3390/cells14181406)
Supplement: Supplementary file 1 [file cells-14-01406-s001.zip › cells-3823415-supplementary.pdf]

Supplementary Table S1. Results of studies reported on oxidative stress markers in different tissues from Friedrich's ataxia (FRDA) patients compared to those of healthy controls (HC)

AAS Atomic absorption spectrophotometry; CAT catalase; Cr creatinine; 2-D-DIGE Two-dimensional difference in-gel electrophoresis; DHBA Dihydroxybenzoic acid; DNA deoxyribonucleic acid; ECLIA Electrochemiluminescence immuno-assay; ELISA Enzyme-Linked ImmunoSorbent Assay; ESI-MS Electrospray ionization-mass spectrometry; F female; FRDA Friedreich's Ataxia; GLRX1 Glutaredoxin 1; GPx glutathione peroxidase; GSH glutathione; GSSG oxidized glutathione; GST glutathione transferase; HC healthy controls; ^{1}H-MRS proton magnetic resonance spectroscopy; 4-HNE 4-hydroxy-2-nonenal; M male; HPLC high performance liquid chromatography; MDA malonyldialdehyde; NAA N-acetyl-aspartate; NO nitric oxide; NSE neuronal specific enolase; OH-dG 8-hydroxy-deoxyguanosine; PGC1-alpha PPARgamma coactivator 1-alpha; ^{31}P-MRS ^{31}phosporus-magnetic resonance imaging; PPARgamma Peroxisome Proliferator Activated Receptor Gamma; RT-PCR real-time polymerase chain reaction; RT-qPCR real-time quantitative PCR; SOD1 superoxide-dismutase 1; SOD2 superoxide-dismutase 2; SWI susceptibility weighted imaging; TAC total antioxidant capacity; TBA thiobarbituric RS thiobarbituric acid reactive substances; TRX1 Thioredoxin 1

BRAIN/SPINAL CORD					
**PARAMETER**	**AUTHOR, YEAR [REF]**	**COUNTRY**	**METHOD**	**STUDY SUBJECTS**	**MAIN FINDINGS**
MDA/TBARS	MacEvilly & Muller, 1997 [6]	United Kingdom	HPLC/fluoro-metric method	4 FRDA patients (M:F 3:1, 24-53 years) and 4 HC (M:F 4:0, 16-61 years)	Non-significant differences in cerebellum, brainstem, and cortex between FRDA patients and HC
In vitro lipid peroxidation susceptibility (MDA)	MacEvilly & Muller, 1997 [6]	United Kingdom	Incubation with $CuSO_4/H_2O_2$. Measurement of MDA by HPLC	3 FRDA patients and 3 HC (age and sex not given)	Significant decrease in brainstem whole homogenates from FRDA patients compared to HC
Glutathione bound to proteins	Sparaco et al., 2009 [7]	Italy	Immunohistoc hemistry	4 FRDA patients (M:F 2:2, 24-47 years) and 10 age-matched HC (age and sex not given)	Significant increase in gray matter neurons as well as in cells and axons of the white matter of the spinal cord from FRDA patients compared to HC
Mitochondrial complex I	Bradley et al., 2000 [8]	United Kingdom	Spectrophoto metry	2 FRDA patients and 2 HC (age and sex not given)	Non-significant differences in cerebellum and dorsal root ganglia
Mitochondrial complex II	Sparaco et al., 2009 [7]	Italy	Immunohistoc hemistry	4 FRDA patients (M:F 2:2, 24-47 years) and 10 age-matched HC (age and sex not given)	Normal expression in the spinal cord from FRDA patients compared to HC
Mitochondrial complex II + III	Bradley et al., 2000 [8]	United Kingdom	Spectrophoto metry	2 FRDA patients and 2 HC (age and sex not given)	Non-significant differences in cerebellum and dorsal root ganglia
Mitochondrial complex IV	Sparaco et al., 2009 [7]	Italy	Immunohistoc hemistry	4 FRDA patients (M:F 2:2, 24-47 years) and 10 age-matched HC (age and sex not given)	Normal expression in the spinal cord from FRDA patients compared to HC
Mitochondrial complex IV	Bradley et al., 2000 [8]	United Kingdom	Spectrophoto metry	2 FRDA patients and 2 HC (age and sex not given)	Non-significant differences in cerebellum and dorsal root ganglia
Aconitase activity	Bradley et al., 2000 [8]	United Kingdom	Spectrophoto metry	2 FRDA patients and 2 HC (age and sex not given)	Non-significant differences in cerebellum and dorsal root ganglia
Iron	Bradley et al., 2000 [8]	United Kingdom	Histochemical analysis	4 FRDA patients (age and sex not given)	No evidence of iron accumulation in cerebellum, spinal cord, dorsal root ganglia, and peripheral nerves

Iron	Solbach et al., 2014 [9]	Germany	Multi-echo gradient echo and susceptibility weighted imaging (SWI) sequences acquired on a 7 T and 1.5 T MRI	14 FRDA patients (M:F 6:8, 38.1 ± 8.5 years, duration of symptoms 17.6 ± 7.2 years) and 14 age-matched HC (H:M 7:7, 38.1 ± 7.7 years)	No evidence of iron accumulation in dentate nuclei from cerebellum despite the presence of cerebellar atrophy
Alpha-tocopherol	MacEvilly & Muller, 1997 [6]	United Kingdom	HPLC	4 FRDA patients (M:F 3:1, 24-53 years) and 4 HC (M:F 4:0, 16-61 years)	Non-significant differences in cerebellum, brainstem, and cortex homogenates between FRDA patients and HC
NAA/Cr (neuroaxonal marker)	Gramegna et al., 2017 [10]	Italy	^{1}H-MRS (left cerebellar hemisphere, including dentate nuclei)	28 FRDA patients (M:F 17:11, 29 ± 12 years) and 35 age-matched HC (M:F 19:16, 35 ± 14 years)	Significant decrease in FRDA patients compared to HC
Cho/Cr (membrane marker)	Gramegna et al., 2017 [10]	Italy	^{1}H-MRS (left cerebellar hemisphere, including dentate nuclei)	28 FRDA patients (M:F 17:11, 29 ± 12 years) and 35 age-matched HC (M:F 19:16, 35 ± 14 years)	Significant decrease in FRDA patients compared to HC
Mean diffusivity	Gramegna et al., 2017 [10]	Italy	^{1}H-MRS (left cerebellar hemisphere, including dentate nuclei)	28 FRDA patients (M:F 17:11, 29 ± 12 years) and 35 age-matched HC (M:F 19:16, 35 ± 14 years)	Significant increase in FRDA patients compared to HC
**PLASMA**					
**PARAMETER**	**AUTHOR, YEAR [REF]**	**COUNTRY**	**METHOD**	**STUDY SUBJECTS**	**MAIN FINDINGS**
MDA	Bradley et al., 2004 [11]	United Kingdom	HPLC followed by spectrophotome try	19 FRDA patients (22.8 ± 7.7 years, disease duration 13.2 ± 6.8 years) and 7 HC (30.4 ± 13.1 years). Sex not given	Significant increase in FRDA patients compared to HC
GSH	Bolotta et al., 2020 [12]	Italy	High-performance capillary electrophoresis	7 FRDA patients (19-26 years, H:M 3:4) and 7 HC (data not given)	Non-significant differences between FRDA patients and HC
GSSG	Bolotta et al., 2020 [12]	Italy	High-performance capillary electrophoresis	7 FRDA patients (19-26 years, H:M 3:4) and 7 HC (data not given)	Significant increase in FRDA patients compared to HC
GSH/GSSG	Bolotta et al., 2020 [12]	Italy	High-performance capillary electrophoresis	7 FRDA patients (19-26 years, H:M 3:4) and 7 HC (data not given)	Significant decrease in FRDA patients compared to HC
Carbonyl proteins	Bolotta et al., 2020 [12]	Italy	Two-dimensional polyacrylamide gel electropho-resis	7 FRDA patients (19-26 years, H:M 3:4) and 7 HC (data not given)	Significant increase in FRDA patients compared to HC

Total antioxidant capacity (TAC)	Bolotta et al., 2020 [12]	Italy	Oxygen radical absorbance capacity (fluorometric) assay	7 FRDA patients (19-26 years, H:M 3:4) and 7 HC (data not given)	Significant decrease in FRDA patients compared to HC
DHBA, a marker of hydroxyl radical attack	Schulz et al, 2000 [13]	Germany and the United States of America	HPLC	33 FRDA patients and 20 age and sex matched HC (age and sex not given)	Non-significant differences between FRDA patients and HC
Iron	Pathak et al., 2019 [14]	India	Nitro PAPS colorimetric assay	25 FRDA patients and 25 age and sex matched HC (age and sex not given)	Significant decrease in FRDA patients compared to HC
Copper	Pathak et al., 2019 [14]	India	Cu-DiBr-PAESA colorimetric assay	25 FRDA patients and 25 age and sex matched HC (age and sex not given)	Significant decrease in FRDA patients compared to HC
Apolipoprotein A-I, C-II, C-III, hemopexin, a1-antitrypsin, complement component C3, transcription factor III-A.	Swarup et al., 2013 [15]	India	Two-dimensional difference in-gel electrophoresis (2D-DIGE) followed by mass spectrometry analysis	42 FRDA patients (17.0 ± 4.0 years, disease duration 7.0 ± 4.0 years) and 22 HC (18.0 ± 3.0 years). Sex not given	Down-regulated in FRDA patients compared to HC
Fibrinogen, antithrombin, antiplasmin, and Albumin	Swarup el al., 2013 [15]	India	Two-dimensional difference in-gel electrophoresis (2D-DIGE) followed by mass spectrometry analysis	42 FRDA patients (17.0 ± 4.0 years, disease duration 7.0 ± 4.0 years) and 22 HC (18.0 ± 3.0 years). Sex not given	Up-regulated in FRDA patients compared to HC
**SERUM**					
**PARAMETER**	**AUTHOR, YEAR [REF]**	**COUNTRY**	**METHOD**	**STUDY SUBJECTS**	**MAIN FINDINGS**
Vitamin E	Barbeau et al., 1984 [16]	Canada	Article not available, quoted by Muller et al.	Article not available, quoted by Muller et al.	Non-significant differences between FRDA patients and controls
Vitamin E and vitamin E/ cholesterol ratio	Muller et al., 1987 [17]	United Kingdom	Colorimetric method	31 FRDA patients (8-59 years, sex not given) and 31 disabled controls (most of them with multiple sclerosis, age and sex not given)	Non-significant differences between FRDA patients and disabled controls
Vitamin E	Eusebi et al., 1990 [18]	Italy	Article not available, reference in PubMed	Article not available, reference in PubMed	Significant decrease in FRDA patients compared to controls
Vitamin E	Cooper et al., 2008 [19]	United Kingdom	Not stated	50 FRDA patients (M:F 21:29, 24.0 ± 10.1 years, disease duration 13.0 ±	Significant decrease in FRDA patients compared to reference values

				8.0 years). Reference laboratory values	
Coenzyme Q_{10}	Cooper et al., 2008 [10]	United Kingdom	Not stated	50 FRDA patients (M:F 21:29, 24.0 ± 10.1 years, disease duration 13.0 ± 8.0 years). Reference laboratory values	Significant decrease in FRDA patients compared to reference values
Uric acid	Schirinzi et al., 2018 [20]	Italy	Indirect uricase ultraviolet method	19 FRDA patients (M:F 9:10, 23.8 ± 15.2 years, disease duration 11.0 ± 6.8 years) and 25 HC (M:F 10:15, 23.8 ± 6.6 years,	Significant increase in FRDA patients compared to reference values. Lack of correlation with disease duration or severity
**WHOLE BLOOD**					
**PARAMETER**	**AUTHOR, YEAR [REF]**	**COUNTRY**	**METHOD**	**STUDY SUBJECTS**	**MAIN FINDINGS**
Frataxin	Lazaropoulos et al., 2015 [21]	United States of America and Canada	Immunoassay	521 FRDA patients (age 28.4 ±15.5 years, M:F 263:258), 306 carriers (47.8 ± 12.8 years, M:F 129:177), and 119 HC (38.1 ± 14.9 years, M:F 63:56)	Non-significant differences between FRDA patients and HC. No changes over time on repeated analyses
Total GSH	Piemonte et al., 2001 [22]	Italy	Reverse phase liquid chromate-graphy with fluorescence detection	14 FRDA patients (M:F 8:6, 8-22 years) and 20 age and sex matched HC (M:F 10:10, age not given)	Non-significant differences between FRDA patients and HC
Total GSH	Petrillo et al., 201 [23]	Italy	Enzymatic cyclic assay	1 FRDA patient, mother and sister (both asymptomatic carriers), and father, and 3 age- and sex-matched controls	Significant decrease in the mother, sister, and father, but not in the proband, compared to controls.
Free GSH	Piemonte et al., 2001 [22]	Italy	Reverse phase liquid chromatography with fluorescence detection	14 FRDA patients (M:F 8:6, 8-22 years) and 20 age and sex matched HC (M:F 10:10, age not given)	Significant decrease in FRDA patients compared to HC
GSSG	Petrillo et al., 201 [23]	Italy	Enzymatic cyclic assay	1 FRDA patient, mother and sister (asymptomatic carrier), and father, and 3 age- and sex-matched controls	Significant decrease in the proband, significant increase in the mother and sister, and similar to that of controls in the father
GPx	McMackin et al., 2019 [24]	United States of America	ELISA commercial kit	5 FRDA patients and 6 controls (data not given)	Significant decrease in FRDA patients compared to HC
**ERYTHROCYTES**					
**PARAMETER**	**AUTHOR, YEAR [REF]**	**COUNTRY**	**METHOD**	**STUDY SUBJECTS**	**MAIN FINDINGS**
Lipid	Bolotta et	Italy	Lipidomic	7 FRDA patients (19-26	Non-significant differences between

peroxidation	al., 2020 [12]		análisis	years, H:M 3:4) and 7 HC (data not given)	FRDA patients and HC
Glutathionyl-Hb	Piemonte et al., 2001 [22]	Italy	Electrospray ionization-mass spectrometry (ESI-MS)	14 FRDA patients (M:F 8:6, 8-22 years) and 20 age and sex matched HC (M:F 10:10, age not given)	Significant decrease in FRDA patients compared to HC
SOD activity	Tozzi et al., 2002 [25]	Italy	Spectrophotome try	14 FRDA patients (M:F 9:5, 8-22 years) and 21 age and sex matched HC (M:F 13:8, age not given)	Significant increase in FRDA patients compared to HC
GPx activity	Tozzi et al., 2002 [25]	Italy	Spectrophotome try	14 FRDA patients (M:F 9:5, 8-22 years) and 21 age and sex matched HC (M:F 13:8, age not given)	Non-significant differences between FRDA patients and HC
GST activity	Tozzi et al., 2002 [25]	Italy	Spectrophotome try	14 FRDA patients (M:F 9:5, 8-22 years) and 21 age and sex matched HC (M:F 13:8, age not given)	Significant increase in FRDA patients compared to HC
Frataxin protein levels	Steinkellne r et al. 2017 [26]	Austria, India, and Germany	Electrochemilu minescence immunoassay (ECLIA)	3 FRDA patients and 3 HC (data not given)	Significant decrease in FRDA patients compared to HC
Iron	Steinkellne r et al. 2017 [26]	Austria, India, and Germany	Atomic absorption spectrophotome try (AAS)	3 FRDA patients and 3 HC (data not given)	Non-significant differences between FRDA patients and HC
Haemoglobin	Steinkellne r et al. 2017 [26]	Austria, India, and Germany	Photometric assay	3 FRDA patients and 3 HC (data not given)	Non-significant differences between FRDA patients and HC
Protoporphyrin IX	Steinkellne r et al. 2017 [26]	Austria, India, and Germany	Autofluores- cence	3 FRDA patients and 3 HC (data not given)	Non-significant differences between FRDA patients and HC
Ferrochelatase	Steinkellne r et al. 2017 [26]	Austria, India, and Germany	Autofluores- cence	3 FRDA patients and 3 HC (data not given)	Non-significant differences between FRDA patients and HC
**LYMPHOCYTES/LYMPHOBLASTS**					
**PARAMETER**	**AUTHOR, YEAR [REF]**	**COUNTRY**	**METHOD**	**STUDY SUBJECTS**	**MAIN FINDINGS**
Mitochondrial complex II	Rötig et al., 1997 [27]	France	Spectrophotome try	2 FRDA patients (compared with data from 68 controls)	Non-significant differences between FRDA patients and controls
Mitochondrial complex III	Rötig et al., 1997 [27]	France	Spectrophotome try	2 FRDA patients (compared with data from 68 controls)	Non-significant differences between FRDA patients and controls
Mitochondrial complex IV	Rötig et al., 1997 [27]	France	Spectrophotome try	2 FRDA patients (compared with data from 68 controls)	Non-significant differences between FRDA patients and controls
Aconitase	Rötig et al., 1997 [27]	France	Spectrophotome try	2 FRDA patients (compared with data from 68 controls)	Non-significant differences between FRDA patients and controls

Total glutathione (mitochondria)	Bulteau et al., 2021 [28]	France	Enzymatic assay	3 FRDA patients and 3 HC (age and sex not given)	Non-significant differences between FRDA patients and HC
GSH/GSSG (mitochondria)	Bulteau et al., 2021 [28]	France	Enzymatic assay	3 FRDA patients and 3 HC (age and sex not given)	Significant decrease in FRDA patients compared to HC
Thiol oxidation/glutathionylation (mitochondria)	Bulteau et al., 2021 [28]	France	Western blot, immunodetection	3 FRDA patients and 3 HC (age and sex not given)	Significant increase for alpha-ketoglutarate and mitochondrial complexes III and IV in FRDA patients compared to HC
**PERIPHERAL BLOOD MONONUCLEAR CELLS**					
**PARAMETER**	**AUTHOR, YEAR [REF]**	**COUNTRY**	**METHOD**	**STUDY SUBJECTS**	**MAIN FINDINGS**
Mitochondrial complex I	Selak et al., 2011 [29]	United States of America	Spectrophotometry	34 FRDA patients (M:F 25:9, mean age 35 years) and 23 age and sex matched HC (M:F 15:8, mean age 32 years)	Non-significant differences between FRDA patients and HC
Mitochondrial complex II	Selak et al., 2011 [29]	United States of America	Spectrophotometry	34 FRDA patients (M:F 25:9, mean age 35 years) and 23 age and sex matched HC (M:F 15:8, mean age 32 years)	Non-significant differences between FRDA patients and HC
Mitochondrial complex IV	Selak et al., 2011 [29]	United States of America	Spectrophotometry	34 FRDA patients (M:F 25:9, mean age 35 years) and 23 age and sex matched HC (M:F 15:8, mean age 32 years)	Non-significant differences between FRDA patients and HC
Frataxin	Selak et al., 2011 [29]	United States of America	Lateral flow (dip-stick) immunoassay	34 FRDA patients (M:F 25:9, mean age 35 years) and 23 age and sex matched HC (M:F 15:8, mean age 32 years)	Significant decrease in mitochondria from FRDA patients compared to HC
SOD1 activity	Selak et al., 2011 [29]	United States of America	Spectrophotometry	34 FRDA patients (M:F 25:9, mean age 35 years) and 23 age and sex matched HC (M:F 15:8, mean age 32 years)	Non-significant differences between FRDA patients and HC
**PLATELETS**					
**PARAMETER**	**AUTHOR, YEAR [REF]**	**COUNTRY**	**METHOD**	**STUDY SUBJECTS**	**MAIN FINDINGS**
Mitochondrial complex I	Selak et al., 2011 [29]	United States of America	Spectrophotometry	34 FRDA patients (M:F 25:9, mean age 35 years) and 23 age and sex matched HC (M:F 15:8, mean age 32 years)	Non-significant differences between FRDA patients and HC
Mitochondrial complex II	Selak et al., 2011 [29]	United States of America	Spectrophotometry	34 FRDA patients (M:F 25:9, mean age 35 years) and 23 age and	Non-significant differences between FRDA patients and HC

				sex matched HC (M:F 15:8, mean age 32 years)	
Mitochondrial complex IV	Selak et al., 2011 [29]	United States of America	Spectrophotometry	34 FRDA patients (M:F 25:9, mean age 35 years) and 23 age and sex matched HC (M:F 15:8, mean age 32 years)	Non-significant differences between FRDA patients and HC
Frataxin	Selak et al., 2011 [29]	United States of America	Lateral flow (dip-stick) immunoassay	34 FRDA patients (M:F 25:9, mean age 35 years) and 23 age and sex matched HC (M:F 15:8, mean age 32 years)	Significant decrease in mitochondria from FRDA patients compared to HC
SOD1 activity	Selak et al., 2011 [29]	United States of America	Spectrophotometry	34 FRDA patients (M:F 25:9, mean age 35 years) and 23 age and sex matched HC (M:F 15:8, mean age 32 years)	Non-significant differences between FRDA patients and HC

SKIN FIBROBLASTS

PARAMETER	AUTHOR, YEAR [REF]	COUNTRY	METHOD	STUDY SUBJECTS	MAIN FINDINGS
4-hydroxynonenal (4-HNE)	Quatrana et al., 2025 [30]	Italy	ELISA method	3 FRDA patients (M:F 2:1, 8-19 years) and 3 age- and sex matched controls	Significant increase in FRDA patients compared to HC
Mitochondrial complex II	Rötig et al., 1997 [27]	France	Spectrophotometry	2 FRDA patients (compared with data from 68 controls)	Non-significant differences between FRDA patients and controls
Mitochondrial complex III	Rötig et al., 1997 [27]	France	Spectrophotometry	2 FRDA patients (compared with data from 68 controls)	Non-significant differences between FRDA patients and controls
Mitochondrial complex IV	Rötig et al., 1997 [27]	France	Spectrophotometry	2 FRDA patients (compared with data from 68 controls)	Non-significant differences between FRDA patients and controls
Aconitase	Rötig et al., 1997 [27]	France	Spectrophotometry	2 FRDA patients (compared with data from 68 controls)	Non-significant differences between FRDA patients and controls
Aconitase	Bradley et al., 2004 [11]	United Kingdom	Spectrophotometry	8 FRDA patients and 4 HC (age and sex not given)	Non-significant decrease in FRDA patients compared to HC
Total GSH	Bradley et al., 2004 [11]	United Kingdom	GSH kit assay	8 FRDA patients and 4 HC (age and sex not given)	Non-significant differences between FRDA patients and HC
Total GSH	Petrillo et al., 201 [31]	Italy	Enzymatic cyclic assay	3 FRDA patients (2 males, 1 female, 8-19 years) and 3 age- and sex-matched controls	Significant decrease in FRDA patients compared to HC
Total GSH	Petrillo et al., 201 [31]	Italy	Enzymatic cyclic assay	1 FRDA patient, mother and sister (both asymptomatic carriers), and father, and 3 age- and sex-matched controls	Significant decrease in the mother and father, but not in the proband and his sister, compared to controls.
GSH/GSSG ratio	Pastore et al., 2003 [32]	Italy	HPLC	9 FRDA patients (M:F 5:4, age not given) and 4 age-	Significant decrease in FRDA patients compared to HC

				matched controls (sex not given)	
Protein-bound/total GSH	Pastore et al., 2003 [32]	Italy	HPLC	9 FRDA patients (M:F 5:4, age not given) and 4 age-matched controls (sex not given)	Significant increase in FRDA patients compared to HC
Protein-bound/free GSH	Pastore et al., 2003 [32]	Italy	HPLC	9 FRDA patients (M:F 5:4, age not given) and 4 age-matched controls (sex not given)	Significant increase in FRDA patients compared to HC
Glutathionylation of actin	Pastore et al., 2003 [32]	Italy	Western blot	9 FRDA patients (M:F 5:4, age not given) and 4 age-matched controls (sex not given)	Significant increase in FRDA patients compared to HC
Susceptibility to oxidative stress	Bradley et al., 2004 [11]	United Kingdom	Incubation with H_2O_2 and with $FeCl_3$	8 FRDA patients and 4 HC (age and sex not given)	Non-significant differences between FRDA patients and HC
Superoxide levels	García-Giménez et al., 2011 [33]	Spain	Fluorescence method	3 FRDA patients (M:F 1:2, 13-30 years, age at onset 8-25 years) and 3 controls (M:F 1:2, 11-50 years)	Significant increase in FRDA patients compared to HC
Frataxin levels	García-Giménez et al., 2011 [33]	Spain	RT-PCR	3 FRDA patients (M:F 1:2, 13-30 years, age at onset 8-25 years) and 3 controls (M:F 1:2, 11-50 years)	Significant decrease of mRNA expression in FRDA patients compared to HC
Frataxin levels	Dey et al., 2012 [34]	United kingdom	RP-PCR and Western blot	FRDA patients and HC (number, age, and sex not given)	Significant decrease of mRNA expression in FRDA patients compared to HC
Frataxin levels	Petrillo et al., 201 [31]	Italy	Enzymatic cyclic assay	3 FRDA patients (2 males, 1 female, 8-19 years) and 3 age- and sex-matched controls	Significant decrease in FRDA patients compared to HC
Cu/Zn-SOD levels	García-Giménez et al., 2011 [33]	Spain	RT-PCR	3 FRDA patients (M:F 1:2, 13-30 years, age at onset 8-25 years) and 3 controls (M:F 1:2, 11-50 years)	Non-significant differences in mRNA expression between FRDA patients and HC, significant decrease of protein levels and activity in FRDA patients compared to HC
Cu/Zn-SOD levels	Dey et al., 2012 [34]	United kingdom	RP-PCR and Western blot	FRDA patients and HC (number, age, and sex not given)	Significant decrease of mRNA expression in FRDA patients compared to HC
Mn-SOD levels	Marmolino et al., [35]	Belgium and Italy	RT-qPCR	5 FRDA patients and 5 HC (age and sex not specified)	Significant decrease of mRNA expression and protein levels in FRDA patients compared to HC
Mn-SOD levels	García-Giménez et al., 2011 [33]	Spain	RT-PCR	3 FRDA patients (M:F 1:2, 13-30 years, age at onset 8-25 years) and 3 controls (M:F 1:2, 11-50 years)	Significant decrease of mRNA expression, protein levels, and activity in FRDA patients compared to HC
Mn-SOD levels	Dey et al., 2012 [34]	United kingdom	RT-PCR and Western blot	FRDA patients and HC (number, age, and sex not given)	Significant decrease of mRNA expression in FRDA patients compared to HC
GPx1 levels	García-Giménez et al., 2011 [33]	Spain	RT-PCR	3 FRDA patients (M:F 1:2, 13-30 years, age at onset 8-25 years) and 3 controls (M:F 1:2, 11-50 years)	Non-significant differences in mRNA expression, protein levels, and activity in FRDA patients compared to HC
CAT levels	García-	Spain	RT-PCR	3 FRDA patients (M:F 1:2,	Non-significant differences in

	Giménez et al., 2011 [33]			13-30 years, age at onset 8-25 years) and 3 controls (M:F 1:2, 11-50 years)	mRNA expression, protein levels, and activity in FRDA patients compared to HC
Thioredoxin 1 (TRX1)	Quatrana et al., 2025 [30]	Italy	ELISA method	3 FRDA patients (M:F 2:1, 8-19 years) and 3 age- and sex matched controls	Significant upregulation in FRDA patients compared to HC, which was associated with activation of Nuclear factor-κB p65 subunit (NF-kB p65)
Glutaredoxin 1 (GLRX1)	Quatrana et al., 2025 [30]	Italy	ELISA method	3 FRDA patients (M:F 2:1, 8-19 years) and 3 age- and sex matched controls	Significant upregulation in FRDA patients compared to HC, which was associated with activation of Nuclear factor-κB p65 subunit (NF-kB p65)
Peroxisome Proliferator Activated Receptor Gamma (PPARgamma) Coactivator 1-alpha (PGC-1alpha) levels	García-Giménez et al., 2011 [33]	Spain	RT-PCR	3 FRDA patients (M:F 1:2, 13-30 years, age at onset 8-25 years) and 3 controls (M:F 1:2, 11-50 years)	Significant increase in mRNA expression in FRDA patients compared to HC
Peroxisome Proliferator Activated Receptor Gamma (PPARgamma) Coactivator 1-alpha (PGC-1alpha) levels	Marmolino et al., [35]	Belgium and Italy	RT-qPCR	5 FRDA patients and 5 HC (age and sex not specified)	Significant decrease in mRNA expression and protein levels in FRDA patients compared to HC
**ENDOMYOCARDIAL TISSUE**					
**PARAMETER**	**AUTHOR, YEAR [REF]**	**COUNTRY**	**METHOD**	**STUDY SUBJECTS**	**MAIN FINDINGS**
Mitochondrial complex I	Rötig et al., 1997 [27]	France	Spectrophotometry	2 FRDA patients (compared with data from 68 controls)	Significantly decreased activity in FRDA patients compared to controls
Mitochondrial complex I	Bradley et al., 2000 [8]	United Kingdom	Spectrophotometry	9 FRDA patients and 5 HC (age and sex not given)	Significantly decreased activity in FRDA patients compared to controls
Mitochondrial complex II	Rötig et al., 1997 [27]	France	Spectrophotometry	2 FRDA patients (compared with data from 68 controls)	Significantly decreased activity compared to controls
Mitochondrial complex III	Rötig et al., 1997 [27]	France	Spectrophotometry	2 FRDA patients (compared with data from 68 controls)	Significantly decreased activity compared to controls
Mitochondrial complex II + III	Bradley et al., 2000 [8]	United Kingdom	Spectrophotometry	9 FRDA patients and 5 HC (age and sex not given)	Significantly decreased activity in FRDA patients compared to controls
Mitochondrial complex IV	Rötig et al., 1997 [27]	France	Spectrophotometry	2 FRDA patients (compared with data from 68 controls)	Non-significant differences between FRDA patients and controls
Mitochondrial complex IV	Bradley et al., 2000 [8]	United Kingdom	Spectrophotometry	9 FRDA patients and 5 HC (age and sex not given)	Non-significant differences between FRDA patients and controls

Mitochondrial complex V	Rötig et al., 1997 [27]	France	Spectrophotometry	2 FRDA patients (compared with data from 68 controls)	Non-significant differences between FRDA patients and controls
Aconitase	Rötig et al., 1997 [27]	France	Spectrophotometry	2 FRDA patients (compared with data from 68 controls)	Significantly decreased activity compared to controls
Aconitase	Bradley et al., 2000 [8]	United Kingdom	Spectrophotometry	9 FRDA patients and 5 HC (age and sex not given)	Significantly decreased activity in FRDA patients compared to controls
Phosphocreatine/ ATP ratio	Lodi et al., 2001 and 2002 [36, 37]	Italy and the United Kingdom	^{31}P-MRS	9 FRDA patients (16-40 years) and 10 HC (22-41 years). Sex not given	Significant decrease in FRDA patients with (n=5) and without (n=4) left ventricular hypertrophy compared to controls
**SKELETAL MUSCLE TISSUE**					
**PARAMETER**	**AUTHOR, YEAR [REF]**	**COUNTRY**	**METHOD**	**STUDY SUBJECTS**	**MAIN FINDINGS**
Mitochondrial complex I	Rötig et al., 1997 [27]	France	Spectrophotometry	2 FRDA patients (compared with data from 68 controls)	Non-significant differences between FRDA patients and controls
Mitochondrial complex I	Bradley et al., 2000 [8]	United Kingdom	Spectrophotometry	5 FRDA patients and 5 HC (age and sex not given)	Non-significant differences between FRDA patients and controls
Mitochondrial complex II	Rötig et al., 1997 [27]	France	Spectrophotometry	2 FRDA patients (compared with data from 68 controls)	Non-significant differences between FRDA patients and controls
Mitochondrial complex III	Rötig et al., 1997 [27]	France	Spectrophotometry	2 FRDA patients (compared with data from 68 controls)	Non-significant differences between FRDA patients and controls
Mitochondrial complex II + III	Bradley et al., 2000 [8]	United Kingdom	Spectrophotometry	5 FRDA patients and 5 HC (age and sex not given)	Non-significant differences between FRDA patients and controls
Mitochondrial complex IV	Bradley et al., 2000 [8]	United Kingdom	Spectrophotometry	5 FRDA patients and 5 HC (age and sex not given)	Non-significant differences between FRDA patients and controls
Aconitase	Rötig et al., 1997 [27]	France	Spectrophotometry	2 FRDA patients (compared with data from 68 controls)	Non-significant differences between FRDA patients and controls
Aconitase	Bradley et al., 2000 [8]	United Kingdom	Spectrophotometry	5 FRDA patients and 5 HC (age and sex not given)	Non-significant differences between FRDA patients and controls
Iron	Bradley et al., 2000 [8]	United Kingdom	Histochemical analysis	4 FRDA patients	No evidence of iron accumulation
Frataxin	Nachbauer et al., 2011 [38]	Austria	Electrochemiluminescence immunoassay	7 FRDA patients (M:F 6:1, 40 $\pm$ 14 years, age at onset 22 $\pm$ 8 years)	Significant decrease compared with PBMCs of the same patients, and correlation between muscle and PBMC levels
Phosphocreatine/ ATP ratio	Lodi et al., 2001 and 2002 [36, 37]	Italy and the United Kingdom	^{31}P-MRS	9 FRDA patients (16-40 years) and 10 HC (22-41 years). Sex not given	Significant decrease in FRDA patients compared to controls
**URINE**					
**PARAMETER**	**AUTHOR, YEAR [REF]**	**COUNTRY**	**METHOD**	**STUDY SUBJECTS**	**MAIN FINDINGS**
8-hydroxy-2'-deoxyguanosine (8OH2'dG),	Schulz et al, 2000 [13]	Germany and the United	HPLC	33 FRDA patients and 20 age and sex matched HC (age and sex not given(	Significant increase in FRDA patients compared to HC

		States of America			
**BUCCAL CELLS**					
**PARAMETER**	**AUTHOR, YEAR [REF]**	**COUNTRY**	**METHOD**	**STUDY SUBJECTS**	**MAIN FINDINGS**
Frataxin	Lazaropoulos et al., 2015 [21]	United States of America and Canada	Immunoassay	521 FRDA patients (age 28.4 $\pm$15.5 years, M:F 263:258), 306 carriers (47.8 $\pm$ 12.8 years, M:F 129:177), and 119 HC (38.1 $\pm$ 14.9 years, M:F 63:56)	Non-significant differences between FRDA patients and HC. No changes over time on repeated analyses

Supplementary Table S2. Results of clinical trials with antioxidant compounds tested in patients with FRDA

ADLS Activities of Daily Living Scale: ATP adenosin-triphosphate; CGIC clinical global impression of change; Cho choline; Cr creatinine; DI Disposition Index; DTI diffusion tensor imaging; DWI diffusion-weighted imaging; EPI-743 alpha-tocotrienolol quinone or vatiquinone; ELISA Enzyme-Linked ImmunoSorbent Assay; EPI (+)-Epicatechin; EPO erythropoietin; FAIS Friedreich Ataxia Impact Scale; FRDA Friedreich ataxia; FARS Friederich Ataxia Rating Scale; fsIVGTT modified, frequently sampled intravenous glucose tolerability test; GAA guanine-adenosine-adenosina; G-CSF granulocyte-colony stimulating factor;

GICS Global Impression of Clinical Severity; GSH reduced glutathione; GSSG oxidized glutathione; 9HPT 9-Hole Peg Test; ICARS International Cooperative Ataxia Rating Scale; IVS interventricular septal; HPLC high performance liquid chromatography; LCSLA Low Contrast Sloan Letter Acuity; LVEF left ventricular ejection fraction; LVM left ventricular mass; LVMI left ventricular mass index; LVPV left ventricular posterior wall; mFARS modified FARS; MFIS modified Fatigue Impact Scale; MRI magnetic resonance imaging; mRNA messenger ribonucleic acid; NAA N-acetyl-aspartate; NRF1 nuclear respiratory factor 1; Nrf2 nuclear factor E2-related factor 2; OH-dG 8-hydroxy-deoxyguanosine, PBMC peripheral blood mononuclear cells; PedsQL Pediatric Quality of Life Inventory 4.0; PGC1α peroxisome proliferator-activated receptor-gamma coactivator 1 alpha; PGIC patient global impression of change; ^{31}phosporus-magnetic resonance imaging; PWT posterior wall thickness RWT relative wall thickness; SARA Scale for the Assessment and Rating of Ataxia; SF36 36-Item Short Form Health Survey; SWT septal wall thickness, T25FW Timed 25 Foot Walk (T25FW)

DRUG	AUTHOR, YEAR [REF]	COUNTRY	STUDY DESIGN	MAIN FINDINGS
Idebenone alone	Rustin et al., 1999 [92]	France	Open-label study involving 3 FRDA patients, with administration of idebenone 5 mg/Kg/day for 5-9 months.<br>Echocardiographic assessment<br>Measurement of mitochondrial complex II and aconitase activities and idebenone oxidation/reduction in endomyocardial biopsy	Significant decrease in septal thickness, left-ventricular wall thickness, and left ventricular mass index by echocardiography.<br>No substantial changes in ataxia, but subjective improvement in strength and delicate movements such as handwriting<br>Reduction in the decrease of complex II activity and in lipid peroxidation induced by reduced iron in myocardial tissue. Good tolerability.
	Schulz et al, 2000 [13]	Germany and the United States of America	27 FRDA patients, 8 of them treated with 5 mg/Kg/day of idebenone<br>Measurement of urinary 8-hydroxy-2'-deoxyguanosine (8OH2'dG) by HPLC	Significant decrease in urine 8OH2'dG levels in the group of patients treated with idebenone. Good tolerability
	Schols et al., 2001 [93]	Germany	Placebo-controlled crossover trial involving 9 FRDA patients (5 males, 4 females), with administration of idebenone 360 mg/day for 6 weeks, with 3 weeks of washout between the 2 phases<br>Assessment of neurological function with the ICARS and a motor performance test<br>Assessment of cardiac function with echocardiography<br>Assessment of recovery of skeletal muscle phosphocreatine after exercise with ^{31}P-MRS	No significant changes in ICARS and motor performance<br>No significant changes in echocardiographic measures<br>No significant improvement of mitochondrial impairment (assessed by measurement of recovery of phosphocreatine in skeletal muscle). Good tolerability

	Hausse et al., 2002 [94]<br>Rustin et al., 2002 [95]	France	Prospective open-label study involving 38 patients with FRDA aged 4–22 years (20 males, 18 females), with administration of idebenone 5 mg/Kg/day for 6 months<br>Echocardiographic assessment	Significant decrease in left ventricular wall thickness by 20% in half of the patients and reduction of shortening fraction in 6 patients. Improvement was not related to the number of GAA repeats in the frataxin gene. Good tolerability
	Artuch et al., 2002 [96]	Spain	Open-label study involving 9 FRDA patients (11-19 years) with administration of idebenone 5 mg/Kg/day for 1 year.<br>Assessment of neurological function with the ICARS and neurophysiological studies<br>Assessment of cardiac function with echocardiography.<br>Measurement of serum idebenone concentrations by HPLC with electrochemical detection.	Significant improvement in ICARS scores after 3 months of therapy that was correlated with serum concentrations of idebenone.<br>No significant changes in echocardiographic measurements<br>No significant changes in EMG and ENG studies<br>Positive correlation between serum idebenone levels and differences in ICARS.<br>Good tolerability
	Mariotti et al., 2003 [97]	Italy	1-year, randomized, placebo-controlled, double-blind trial involving 29 FRDA patients receiving 5 mg/Kg/day of idebenone (11 males, 3 females, age 26.1$\pm$1.6 years) or placebo (12 males, 3 females, age 26.3$\pm$2.6 years)<br>Assessment of interventricular septal (IVS) thickness, left ventricular posterior wall (LVPW), and left ventricular mass (LVM) by echocardiography<br>Assessment of neurological function with the ICARS	Significant differences in IVS thickness, LVPW, and LVM between patients treated with idebenone and placebo related to a modest improvement in patients under idebenone and worsening in patients under placebo.<br>No significant changes in ICARS scores.<br>Good tolerability
	Buyse et al., 2003 [98]	Belgium	Prospective open-label study involving 8 patients with FRDA with hypertrophic cardiomyopathy aged 8.6-27.1 years with administration of idebenone 5 mg/Kg/day for 1 year<br>Assessment of LV mass index (LVMI) and LV ejection fraction (LVEF) by echocardiography<br>Assessment of neurological function by the Cooperative Ataxia Group (CAG) Rating Scale for Drug Trial for FRDA<br>Assessment of erythrocyte protoporphyrin concentration by fluorometry	Significant improvement in LVMI in 6 of 8 patients. Non-significant changes in LVEF<br>Progressive worsening in the ataxia rating scale<br>Reduction in erythrocyte protoporphyrin IX levels in five of six patients with elevated baseline levels (unrelated to cardiac improvement)<br>Good tolerability
	Ribaï et al. 2007 [99]	France	Prospective follow-up of 104 FRDA patients (age 36 $\pm$ 11 years, age at onset 16 $\pm$ 11 years, sex not given) during 5 (0.5-7) years. 88 of them received idebenone 5 mg/Kg/day, and 16 preferred not to be treated<br>Neurological assessment with the ICARS and a quantitative writing test to evaluate cerebellar ataxia<br>Cardiac evaluation (70 patients) with echocardiography: thicknesses of the left ventricular septum and posterior wall, and the diameter of the left ventricle at the end of	Progressive increase in ICARS and in the quantitative writing test despite idebenone treatment<br>Presence of square wave jerks in 35/37 at baseline and in 37/37 at the end of follow-up in electro-oculographic studies<br>Significant reduction in left ventricular mass index and posterior wall thickness, but not in septal wall thickness

			diastole and at the end of systole<br>Oculomotor function assessment by electrooculography (37 patients)	Good tolerability
	Prospero et al., 2007 [100]	United States of America	6-month, prospective, randomised, double-blind, placebo-controlled study involving 47 FRDA patients aged 9-17 years<br>Administration of placebo (n=11) or one of 3 doses of idebenone (~5 mg/kg, 15 mg/kg, and 45 mg/kg), stratified by body weight (n = 11, 13, and 12, respectively)<br>Neurological assessment with the ICARS, the FARS, and an ADL scale<br>Determinations of urinary 8OH2´dG using carbon column-based liquid chromatography with electrochemical detection	Lack of significant changes in ICARS, FARS, and ADL scales in the whole series or patients under idebenone, but dose-related improvement for intermediate and high doses.<br>Good tolerability for the 3 doses of idebenone<br>Non-significant reduction of urinary 8OH2´dG levels
	Pineda et al., 2008 [101]	Spain	3-5 years, open-labelled prospective study involving 10 FRDA paediatric patients (8–18 years) and 14 FRDA adult patients (18–46 years)<br>Administration of idebenone (5–20mg/kg/day) for 3–5 years.<br>Neurological assessment with the ICARS<br>Cardiological assessment using echocardiography: fractional shortening (FS), ejection fraction (EF), septum (SP) and posterior wall (PW) thickness, and left ventricular mass index (LVMI)<br>Determinations of plasma idebenone levels by HPLC with electrochemical detection and concentrations of other antioxidants (tocopherol, retinol, coenzyme Q10, selenium, zinc, antioxidant enzymes in erythrocytes – SOD, CAT, GPx, GR) and plasma MDA by standardized methods	Non-significant changes in ICARS in pediatric patients, and a significant increase in adult patients<br>Non-significant changes in echocardiographic parameters were observed in neither pediatric nor adult patients.<br>Non-significant changes in biochemical parameters.<br>Good tolerability
	Rinaldi et al., 2009 [102]	Italy	5-year retrospective analysis of a cohort of 35 FRDA patients treated with idebenone 5 mg/kg/day<br>Neurological assessment by the ICARS<br>Cardiological assessment using echocardiography: interventricular septum (IVS) and posterior wall (PW) thickness and left ventricular (LV) ejection fraction (EF)	Significant increase in ICARS during the follow-up<br>Significant increase in interventricular septum and posterior wall thickness in the group without left ventricular hypertrophy before treatment, and no change in the group with left ventricular hypertrophy<br>Good tolerability
	Brandsema et al., 2010 [103]	Canada	1 year, prospective observational study involving 7 FRDA patients (4 males, 3 females; age 15.87 ± 2.21, age at onset 8.5 ± 2.78 years) treated with idebenone 20 mg/Kg/day<br>Neurological evaluation with the ICARS, Pediatric Quality of Life Inventory 4.0 (PedsQL), and Activities of Daily Living Scale(ADLS)	Non-significant changes in ICARS and ADLS scores.<br>Significant worsening of the physical component but improvement in the emotional, social, and school components of PedsQL<br>Good tolerability
	Lynch et al., 2010 [104]	United States of America	6-month, double-blind, randomized, placebo-controlled, parallel-group study involving 70 FRDA patients aged 8-18 years.<br>Randomization to idebenone 450-900 mg/day	Non-significant differences in ICARS, FARS, and ADLS scores between patients under idebenone or placebo (despite

			(n=22), idebenone 1350-2250 mg/day (n=24) depending on weight, or placebo (n=24)<br>Neurological evaluation with ICARS, FARS, and ADLS	slight improvement of ICARS and FARS with idebenone and slight worsening with placebo)<br>Good tolerability
	Lagedrost et al., 2011 [105]	United States of America	6-month, double-blind, randomized, placebo-controlled, parallel-group study involving 70 FRDA patients aged 8-18 years.<br>Randomization to idebenone 450-900 mg/day (n=22), idebenone 1350-2250 mg/day (n=24) depending on weight, or placebo (n=24)<br>Cardiological evaluation with EKG and echocardiography: left ventricular mass index (LVMI). **relative wall thickness** (RWT), interventricular septum (IVS) and posterior wall (PW) thickness, and left ventricular (LV) ejection fraction (EF)	90% of FRDA patients have EKG abnormalities, and 81.4% left ventricular hypertrophy at baseline.<br>Non-significant effect of idebenone on EKG or echocardiographic measures<br>Good tolerability
	Meier et al., 2012 [106]	United States of America	12-month, open-label extension study involving 68 FRDA patients who participated in a previous 6-month double-blind randomized study [66, 69]. All patients received idebenone 1350-2250 mg/day, depending on weight<br>Neurological evaluation with ICARS and FARS, and ADLS	Slight worsening in ICARS and FARS scores during the 12 months of the open-label extension study<br>Patients initially randomized to idebenone 1350-2250 mg/day had lower ICARS and FARS scores at the end of the extension study (improvement was higher when excluding posture and stand subscores)<br>Good tolerability
	Cook et al., 2019 [107]	United Kingdom, Germany, Austria, The Netherlands	12-month, double-blind, randomized, placebo-controlled, parallel-group, multicentre withdrawal study involving patients who had already received continuous high-dose idebenone (1350 mg/d if ≤45 kg or 2250 mg/d if >45 kg) in the open-label MICONOS Extension Study<br>This study involved 29 FRDA patients, 16 were assigned to idebenone continuation (9 males, 7 females, age 35.8 ± 16.9 years), and 13 to placebo (9 males, 4 females, age 38.7 ± 16.5 years)<br>Neurological assessment with the ICARS, modified Fatigue Impact Scale (MFIS), 9HPT, clinical global impression of change (CGI-C), and AIDS speech capability test.	Non-significant changes in total ICARS (except improvement when ambulatory patients were analyzed separately), MFIS, 9HPT, and CGIC.<br>Improvement in the AIDS speech capability test of patients under idebenone<br>Good tolerability
Idebenone and deferiprone	Velasco-Sánchez et al., 2011 [108]	Spain	11-month, prospective open-label single-arm study involving 20 FRDA patients (8 males, 12 females, mean age 16.5 years)<br>Administration deferiprone (20 mg/kg/day) as add-on therapy with idebenone (20 mg/kg/day)<br>Neurological assessment with ICARS<br>Cardiological assessment with echocardiography: (fractional shortening (FS), ejection fraction (EF), septum (IVS) and posterior wall (PW) thickness, and left ventricular mass index (LVMI)<br>Assessment of brain iron deposits in the	Non-significant changes in total ICARS score, but worsening in posture and gait subscale, and improvement in kinetic function scores.<br>Significant reduction in IVS and LVMI, and lack of changes in the other echocardiographic measures<br>Significant improvement in iron deposits in the dentate nucleus<br>Good tolerability

			dentate nucleus by MRI for 11 months.	
	Elincx-Benizri et al., 2016 [109]	Israel	6-month randomized, double-blind, placebo-controlled study and further open-label extension study during 10-24 months involving 5 FRDA patients<br>Treatment with deferiprone (20 mg/kg/day) added to idebenone<br>Neurological assessment with FARS, SARA, 9HPT, and SF36 health survey<br>Cardiological assessment with echocardiography (left ventricular hypertrophy)	Improvement in neurological function in 3 patients<br>Improvement in cardiac hypertrophy<br>Good tolerability to medications
Idebenone, deferiprone, and rivoflavin	Arpa et al., 2014 [110]	Spain	15-45 months, prospective open-label single-arm study involving 13 FRDA patients (10 males and 3 females, 30.2 ± 12.1 years)<br>Administration of deferiprone 5-25 mg/kg/day, idebenone at 10-20 mg/kg/day, and riboflavin at 10-15 mg/kg/day.<br>Neurological assessment with SARA and SF-36v2<br>Cardiac assessment with echocardiography: septal wall thickness (SWT) and posterior wall thickness (PWT),<br>left ventricular shortening fraction (LVSF), left ventricular ejection fraction (LVEF), and left ventricular mass<br>index (LVMi)	Better annual worsening rate in SARA scores than a cohort of FRDA patients of the same group. Non-significant changes in SR-36v2<br>Slight decrease in LVMi and stability of LVEF<br>4 discontinuations related to the adverse effects of deferiprone
CoQ_{10} and vitamin E	Lodi et al., 2001 [36]	Italy and the United Kingdom	6-month, open-label study involving 10 FRDA patients (5 males, 38 ± 6 years)<br>Administration of CoQ_{10} (400 mg/day) and vitamin E (2,100 IU/day)<br>Neurological assessment with ICARS<br>Cardiac function assessment with echocardiography (LVMi and EF)<br>Cardiac and skeletal muscle metabolism assessed by ^{31}P-MRS (determination of phosphocreatine and ATP)	Non-significant changes in total ICARS scores, but improvement in the posture and gait subscale<br>Non-significant changes in echocardiographic measures<br>Significant increase in cardiac phosphocreatine/ATP ratio and in skeletal muscle V_{max} (a marker of ATP synthesis) at 3 and 6 months of therapy<br>Good tolerability
	Cooper et al., 2008 [19]	United Kingdom	2-year double-blind randomized trial involving 50 FRDA patients (M:F 21:29, 24.0 ± 10.1 years, disease duration 13.0 ± 8.0 years)<br>Randomization to high dose (CoQ_{10} 600 mg/day and vitamin E 2,100 IU/day, n =24) or low dose (CoQ_{10} 30 mg/day and vitamin E 4 IU/day, n = 26)<br>Neurological evaluation with the ICARS<br>Cardiac evaluation with echocardiography: posterior wall thickness in diastole, intraventricular septal thickness (IVS), and fraction shortening	Non-significant differences in ICARS or in echocardiographic measures in high doses compared to low doses of antioxidant therapy<br>Good tolerability
	Hart et al., 2005 [111]	Italy and the United Kingdom	47-month, open-label study involving 77 FRDA patients (10-57 years)<br>Administration of CoQ_{10} (400 mg/day) and vitamin E (2,100 IU/day)<br>Neurological assessment with ICARS<br>Cardiac function assessment with	Non-significant changes in total ICARS scores, but improvement in the posture and gait subscale<br>Non-significant changes in echocardiographic measures<br>Significant increase in cardiac

			echocardiography (LVMi and EF)<br>Cardiac and skeletal muscle metabolism assessed by ^{31}P-MRS (determination of phosphocreatine and ATP in 10 patients, 5 males and 5 females, 38 ± 6 years)	phosphocreatine/ATP ratio and in skeletal muscle V_{mas} (a marker of ATP synthesis) at 3 and 6 months of therapy
A0001 (α-tocopheryl quinone)	Lynch et al., 2012 [112]	United States of America	21-day, double-blind, randomized, placebo-controlled trial of 2 doses of A0001 involving 31 adults with FRDA<br>Administration of placebo n=10), low-dose A0001 (510 Mg, n=10), or high-dose A0001 (750 mg, n=11)<br>Neurological assessment with FARS, Timed 25 Foot Walk (T25FW), 9HPT, Low Contrast Sloan Letter Acuity (LCSLA), Global Impression of Clinical Severity (GICS), MFIS, ADLs, and SF-36<br>Measurement of change in Disposition Index (DI) from a modified, frequently sampled intravenous glucose tolerability test (fsIVGTT)	Significant dose-related improvement in FARS score<br>Significant improvement in GICS but not in the other clinical scales<br>Non-significant changes in DI<br>Increase in plasma CoQ_{10} levels and no effect on plasma vitamin E levels and mitochondrial complex I activity in lymphocytes<br>Good tolerability for the treatment
EPI-743 (alpha-tocotrienolol quinone; vatiquinone)	Zesiewiciz et al., 2018 [113]	United States of America	Randomized, double-blind, and placebo-controlled trial involving 63 adults with FRDA.<br>First phase of 6 months with randomization to placebo (n=21), 200 mg of EPI-743 (n=22), or 400 mg of EPI-743 (n=21), second phase of 6 months open-label extension in which patients initially on placebo were crossed over to EPI-743 200 mg or 400 mg treatment, and 12 months of open-label extension with all participants receiving 400 mg<br>Neurological assessment with FARS, low-contrast visual acuity, T25FW, and 9HPT<br>Cardiological assessment with echocardiography	21 patients withdrew from the study for reasons not related to important side effects<br>Non-significant changes in FARS, low-contrast visual acuity, T25FW, 9HPT and echocardiographic measures between placebo and EPI-743 at 6 months<br>Significant improvement of FARS of patients who completed the 24 months under EPI-743 compared with an age-, stage- and sex-matched cohort followed as part of a disease natural history database
OXI-3 (tocotrienol mixture) plus idebenone	Bolotta et al., 2020 [12]	Italy	12 months, open-label study involving 7 adult FRDA patients (2 males, 5 females)<br>Administration of OXI-3 (5 mg/Kg/day) and idebenone (5 mg/Kg/day)<br>Neurological assessment with the ICARS<br>Neuroimaging assessment with MRI, brain ^{1}HMRS, diffusion-weighted imaging (DWI), diffusion tensor imaging (DTI), Cardiological assessment by MRI<br>Performance of muscle ^{31}PMRS<br>Biochemical measures, including plasma GSH, GSSG, and carbonyl proteins, lipidomic analyses in erythrocytes, and gene expression in lymphocytes<br>.	Slight worsening of ICARS scores<br>Slight non-significant improvement of ejection fraction in cardiac MRI<br>Non-significant changes in brain ^{1}HMRS, diffusion-weighted imaging (DWI), diffusion tensor imaging (DTI), and in muscle ^{31}PMRS<br>Significant increase in GSH/GSSG ratio and total antioxidant capacity, and decrease in protein carbonyls<br>Non-significant changes in lipid peroxide levels<br>Significant decrease in the *SOD1* and increase in *HAMP* (*hepcidine antimicrobial peptide*) gene expression<br>Good tolerability to tocotrienol
Erythropoiet	Boesch et al.,	Austria	6-month open-label clinical pilot study	Significant improvement in

in (EPO)	2008 [114]		involving 8 adult FRDA patients (5 males, 3 females)<br>Administration of 2.000 IU recombinant human EPO twice a week subcutaneously.<br>Neurological assessment with FARS and SARA<br>Clinical outcome measures included the Ataxia Rating Scales. Measurement of frataxin levels in lymphocytes (chemiluminescence) and urine 8-OHdG levels (ELISA) and serum peroxide levels (peroxide activity assay), and monitoring of hematological parameters	FARS and SARA scores<br>Significant increase in serum frataxin levels<br>Significant decrease in urine 8OHdG and serum peroxide levels<br>Increase in haematocrit in 4 patients requiring phlebotomies
	Mariotti et al., 2012 [115]	Italy	6-month, randomized, placebo-controlled, double-blind, dose-response pilot trial involving 16 adult FRDA patients, all under idebenone (5 mg/Kg/day) treatment<br>Administration of recombinant human erythropoietin intravenously (scaling-up phase: 20,000 IU every 3 weeks, 40,000 IU every 3 weeks, and 40,000 IU every 2 weeks; n = 11) or placebo (n = 15)<br>Neurological assessment with SARA, 9-Hole Peg Test (9HPT), and health related quality-of-life questionnaire (SF-36)<br>Measurement of lymphocyte and serum frataxin levels with ELISA	Non-significant changes in SARA scores, 9HPT, and the SF-36 scale<br>Non-significant changes in lymphocytes and frataxin levels<br>Non-significant changes in haemoglobin concentrations<br>Good tolerability
	Boesch et al., 2014 [116]	Austria, Italy, Germany, and Denmark	103-day, multicenter, double-blind, placebo-controlled, phase II clinical trial involving 36 ambulatory FRDA patients (27.8 $\pm$ 8.7 years)<br>Administration of carbamylated EPO (CEPO) 325 μg thrice-weekly (n=24) or placebo (n=12)<br>Neurological assessment with SARA, FARS, and clinical global impression (CGI)<br>Measurement of buccal cells frataxin levels (ELISA), serum 8-OHdG (ELISA), serum peroxides (peroxide activity assay), and serum MDA (colorimetric assay)	Non-significant changes in SARA, FARS, and CGI scores<br>Non-significant changes in frataxin, 8-OHdG, peroxides, and MDA levels<br>Good tolerability
Resveratrol	Yiu et al., 2015 [117]	Australia, Austria, and China	12-week, open-label, non-randomized, proof-of-principle study involving 24 FRDA patients (16 males, 8 females, mean disease duration 19.3 years)<br>Administration of resveratrol 1 mg/day (n=12) or 5 mg/day (n=12)<br>Neurological assessment with FARS and ICARS, audiologic and speech function, and quality of life scales (Friedreich Ataxia Impact Scale -FAIS- and SF36)<br>Cardiological assessment with echocardiography<br>Measurement of frataxin levels (electrochemiluminescence assay) and mRNA expression in PBMC, and oxidative stress markers (plasma F2-isoprostane and urinary 8-OHdG levels)	Good tolerability to resveratrol.<br>Significant improvement in FARS, ICARS, and speech perception in the 5 mg/day group.<br>Non-significant changes in speech rate, pitch control, voice quality, FAIS, SF-36, and echocardiographic measurements (left ventricular end-diastolic diameter, LVMi, relative wall thickness, ejection fraction)<br>Non-significant changes in PBMC frataxin and mRNA frataxin levels and in urinary 8-OHdG<br>Significant decrease of plasma F2-isoprostane levels in the 5 mg/day group

Omaveloxolone (Nrf2 activator)	Lynch et al., 2021 [118]	United States of America, Italy, the United Kingdom, Austria, and Australia	48-week, double-blind, randomized, placebo-controlled, parallel-group, registrational phase 2 trial involving 103 adult FRDA patients (55 male, 48 female)<br>Administration of omaveloxolone 150 mg/day (n=51) or placebo (n=52)<br>Neurological assessment with modified FARS (mFARS), FA-ADL, PGIC, CGIC, 9HPT, T25-FW, and frequency of falls	Significant improvement in mFARS and in the FA-ADL scale in the omaveloxolone group<br>Non-significant changes in PGIC and CGIC, 9HPT, T25-FW, and frequency of falls<br>Transient reversible increases in aminotransferase, headache, nausea, and fatigue were more common among patients receiving omaveloxolone
(+)-Epicatechin (EPI)	Qureshi et al., 2021 [119]	United States of America	24-week, phase II, open-label, baseline-controlled single-center trial including 10 FRDA patients (10-22 years)<br>Administration of (+)-EPI orally (75 mg/day, with escalation to 150 mg/day at 12 weeks for subjects not showing improvement)<br>Neurological assessment with FARS, 9HPT, and ADL scale<br>Measurement of changes in spinal-cord fractional anisotropy, by axial diffusion tensor imaging (DTI) at levels C1, T1, and L1.<br>Measurement of cerebellar metabolites by 3D MR spectroscopy (3D MRS) over the posterior fossa and the upper cervical cord.<br>Cardiological assessments (left ventricular structure and function) with cardiac MRI and echocardiogram	Non-significant changes in FARS, 9HPT, and ADL<br>Non-significant changes in spinal-cord fractional anisotropy<br>Significant reduction in cerebellar atrophy without changes in cerebellar metabolites (NAA/Cr and Cho/Cr ratios)<br>Significant reduction in LVMI, increase in LVEF, and maximal septal thickness<br>Good tolerability (transient increase in migraine-associated symptoms in 3 patients)
Recombinant human granulocyte-colony stimulating factor (G-CSF)	Kemp et al., 2022 [120]	United Kingdom	Open-label, single-site pilot study involving 7 FRDA patients (3 males, 4 females, 22-64 years)<br>Administration of C-GSF 1.28 million units/Kg/day for 5 days, biochemical determinations over 19 days<br>Measurement of frataxin protein levels in PBMC and platelets by an immunologic assay<br>Analysis of mitochondrial complex II (spectrophotometry) and aconitase (colorimetric assay) activities in PBMC<br>Measurement of immunoreactivity to nuclear respiratory factor 1 (NRF1), nuclear factor E2-related factor 2 (Nrf2), and peroxisome proliferator-activated receptor-gamma coactivator 1 alpha (PGC-1α) in PBMC	Good tolerability to G-CSF (1 withdrawal by nausea)<br>Increase in PBMC and platelet frataxin protein levels through the study period.<br>Increase in mitochondrial complex II and aconitase activities in PBMC through the study period<br>Increase in Nrf2 and PGC-1α expression in PBMC, and lack of significant changes in NRF1 expression<br>Non-serious side effects (one withdrawal because of nausea)
